# Employing bioactive compounds derived from *Ipomoea obscura* (L.) to evaluate potential inhibitor for SARS‐CoV‐2 main protease and ACE2 protein

**DOI:** 10.1002/fft2.29

**Published:** 2020-07-06

**Authors:** Saravana Prabha Poochi, Murugesh Easwaran, Balamuralikrishnan Balasubramanian, Mohan Anbuselvam, Arun Meyyazhagan, Sungkwon Park, Haripriya Kuchi Bhotla, Jeeva Anbuselvam, Vijaya Anand Arumugam, Sasikala Keshavarao, Gopalakrishnan Velliyur Kanniyappan, Manikantan Pappusamy, Tanushri Kaul

**Affiliations:** ^1^ Department of Biochemistry and Bioinformatics Karpagam Academy of Higher Education Coimbatore 641021 India; ^2^ Nutritional Improvement of Crops International Centre for Genetic Engineering and Biotechnology New Delhi 110067 India; ^3^ Department of Food Science and Biotechnology College of Life Science Sejong University Seoul 05006 Republic of Korea; ^4^ Department of Biotechnology Selvamm College of Arts and Science Namakkal 637003 India; ^5^ Euroespes Biomedical Research Centre International Centre of Neuroscience and Genomic Medicine Corunna 15165 Spain; ^6^ Department of Life Sciences Christ (Deemed to be University) Bangalore 560029 India; ^7^ Department of Medicine Section of Internal and Cardiovascular Medicine University of Perugia Perugia Italy; ^8^ Department of Animal Science Bharathidasan University Tiruchirappalli 620024 India; ^9^ Medical Genetics and Epigenetics Laboratory Department of Human Genetics and Molecular Biology Bharathiar University Coimbatore Tamil Nadu India; ^10^ Professor and Emeritus (Rtd.) Human Genetics Laboratory Department of Zoology School of Life Sciences Bharathiar University Coimbatore Tamil Nadu 46 India; ^11^ Department of Chemistry College of Natural and Computational Sciences Aksum University Ethiopia

**Keywords:** ADME, GC‐MS, *Ipomoea Obscura* (L.), Molecular docking, SARS‐CoV‐2

## Abstract

Angiotensin converting enzyme 2 (ACE2) and main protease (M^Pro^) are significant target proteins, mainly involved in the attachment of viral genome to host cells and aid in replication of severe acute respiratory syndrome‐coronaviruses or SARS‐CoV genome. In the present study, we identified 11 potent bioactive compounds from ethanolic leaf extract of *Ipomoea obscura* (L.) by using GC‐MS analysis. These potential bioactive compounds were considered for molecular docking studies against ACE2 and M^Pro^ target proteins to determine the antiviral effects against SARS‐COV. Results exhibits that among 11 compounds from *I. obscura* (L.), urso‐deoxycholic acid, demeclocycline, tetracycline, chlorotetracycline, and ethyl iso‐allocholate had potential viral inhibitory activity. Hence, the present findings suggested that chemical constitution present in *I. obscura* (L.) will address inhibition of corona viral replication in host cells.

## 1 INTRODUCTION

Convolvulaceae is one of the biggest family of flowering plants and possesses many members with medicinal and economic benefits. It comprises of 59 genera and 1,600 species. This family is widely distributed in both tropical and moderate temperature regions. One of its members *Ipomoea obscura* (L.) is a little climbing plant with small heart‐shaped leaves and sharp‐edged apex (Aiping et al., 2020; Saravana Prabha et al., 2017). It is native to parts of Africa, Asia, and certain Pacific Islands. Corolla is comprised of five fully fused petals. Seeds and fruits have been used as a cleansing agent to reduce breathing difficulties, alleviate pain, and improve vision (Shahina, 1994). The phytochemical constituents of *I. obscura* promote anti‐inflammatory activity, antioxidant activity, anticancer activity, and antimicrobial activity against certain microorganisms. Ayurveda has explored numerous medicinal properties of this plant against dysentery, ulcers, hemorrhoids, and swellings (Christophe & Pharm, 2002). This plant grows mainly on fences or low ground cover as substrate in disturbed areas.

Severe acute respiratory syndrome (SARS) is a perilous pulmonary infection triggered by single stranded (+) sense RNA virus. Coronaviruses (CoVs) are largest family of RNA viruses with size ranging from 60 to 140 nm. It is further classified into four genera as α, β, γ, and δ. SARS‐CoV‐2 falls under the genus of β coronavirus (Markus et al., 2019). The main pathways of COVID‐19 infection in humans are through droplets, aerosols, feces, and mouth mucus membranes of infected person and cause adverse symptoms such as typical acute respiratory infection, cough, fever, myalgia, sneezing, acute kidney injury, fatigue, acute liver injury, diarrhea, a sore throat, breathing collapse, as well as vomiting (Anthony & Stanley, 2015). The genome of corona virus composed of nonstructural proteins, accessory proteins, and structural proteins. Structural proteins had four major parts: one is spike surface glycol protein (S), tiny envelope protein (E), matrix protein (M), and nucleo‐capsid protein. The corona virus protein is mainly responsible for entry of SARS‐CoV‐2 in to host cell (Yong & Edward, 2020). Angiotensin converting enzyme 2 (ACE2) is an integral membrane glycoprotein mainly present in kidney, endothelium, lungs, and heart. The receptor‐binding domain (RBD) of spike protein includes important amino acids (L455, F486, Q493, S494, N501, and Y505) that were involved in interaction and communication of single stranded RNA coronavirus genome (Andersen, Rambaut, Lipkin, Holmes, & Garry, 2020; Yifei, 2020). So far, six RBD amino acids have shown best binding activities against ACE2 receptors and for determining the host tropism of SARS‐CoV‐like virus. The spike envelope glycoprotein interacts with cellular receptors ACE2 to initiate membrane fusion and viral replications after the proteolytic cleavage event. Subsequently, viral genome is released into the cytoplasm and replicated viral particles then initiate the viral replication and cause adverse effect on a host organism. By blocking the ACE2/SARS‐CoV‐2 interaction, we could cease the viral replication and multiplication. The ACE2 and spike glycol protein may be considered as the attractive drug target for the discovery and development of effective antiviral drug against the viral disease (Bui et al., 2020; Fatima & Florian, 2020; Othman et al., 2020). One of the best characterized drug targets among CoV is the main protease (M^pro^). Besides the papain‐like protease, this enzyme is necessarily aimed at the dispensation of polyproteins that endure translated since the viral RNA. Impeding the action of this enzyme would block viral replication. Subsequently, proteases reported from Homo sapiens do not cleave the functional site by hybrid analogues, which is yet to be discovered. (Fatima & Florian, 2020; Mothay & Ramesh, 2020; Macchiagodena, Pagliai, & Procacci, 2020; Nisha Muralidharan, Sakthivel, Velmurugan, & Michael Gromiha, 2020). At present, there are no effective inhibitors against the novel corona virus; therefore, developing protein‐based inhibitors for corona virus may emerge as a prerequisite strategy for curbing this virus. Computational methodologies have become an essential tool for drug discovery programs for the identification to lead optimization and formulation. Approaches such as ligand or structural based in silico techniques are commonly used in many discovery efforts. Henceforth, present investigation is carried out to identify bioactive compounds from *I. obscura* (L) and examine its antiviral activity against COVID‐19 by the in silico approach.

## 2 MATERIALS AND METHODS

### 2.1 Sample leaves collection and hydrodistillation

The healthy mature and fresh leaves of *I. obscura* (L.) Ker Gawl were collected from Madurai district of Tamil Nadu, India. Taxonomic survey was authenticated by Dr. G.V.S. Murthy, Botanical Survey of India, TNAU Campus, Coimbatore, India. The documented specimen was deposited at TNAU Botany laboratory and utilized for further investigation. All leaf samples were maintained in our laboratory at proper environmental conditions and further used for phytochemical analysis.

### 2.2 Chemicals reagents and apparatus

All chemical reagents were purchased from Sigma‐Aldrich and applied for analysis with no further purification. The major apparatus included a hydrodistillation apparatus, a polarimeter, a Jasco V630 spectrophotometer, and a gas chromatography‐mass spectrometer (GC−MS).

### 2.3 Ethanolic extract preparation

The fresh leaves of *I. obscura* (L.) were carefully washed in running tap water, shade dried for 1 week, and powdered in an electric mixer grinder. The powdered leaves were subjected to ethanol solvent extraction. In total 300 g of dried plant powder was extracted utilizing Soxhlet extraction with 1.5 L of ethanol in a random shaker for 72 h at room temperature. Solvent extract was then evaporated using a rotary evaporator. The dried extract was collected in airtight bottles and stored at 4°C for further studies.

### 2.4 Phytochemical analysis

Based on the preliminary phytochemical screening, the ethanolic leaf extract of *I. obscura* (L.) was subjected to GC‐MS analysis on a Perkin Elmer gas chromatograph Clarus 500 Perkin Elmer system comprising an AOC‐20i auto sampler and a gas chromatograph interfaced to a mass spectrometer prepared with Elite 5MS (5% diphenyl/95% dimethyl polysiloxane) fused a silica column (30 mm × 0.25 mm × 0.25 μm df) that operated in electron impact mode with an ionization energy at 70 ev. Helium gas (99.999%) was utilized as a carrier gas at a constant flow rate of 0.1 mL/min and volume of 2 L was analyzed. The injector temperature was maintained at constant 250°C. Compounds present in ethanolic leaf extract were detected and cross‐checked by comparing their retention indices and mass spectra fragmentation patterns using stored database in a computer library and with published literature, NIST08s.LIB (Lafferly, 1989) and WILEY8.LIB (Stein, 1990).

### 2.5 Molecular docking simulation

Molecular docking is a computational approach for scrutinizing the interaction between the therapeutic target and a small molecule. In this investigation we performed in silico docking by applying Glide 5.5 (Dik‐Lung, Daniel, & Chung, 2011; Glide, 2009), against respiratory therapeutic target ACE2 exhibited in human and M^Pro^ in SARS‐CoV‐2. The in silico docking exploration involves five steps, which are as follows.

#### 2.5.1 Collection and preparation of therapeutic target proteins

PDB Protein retrieval: The therapeutic targets ACE2 (PDB: 6M0J) and M^Pro^ (PDB: 6LU7) were retrieved from PDB structure, which possessed a resolution value 2.16 and 2.45 Å, respectively (Jin et al., 2020; Lan et al., 2020). Target proteins were preprocessed such as refinement, assigning bond orders, treating metals, and treating disulfides, building missing heavy atoms, formal charges, adjusting bond orders, adding hydrogen atom, and undesirable water molecule using PP‐wizard. The protein structure energy was minimized until root mean square deviation (RMSD) cutoff was touched 0.30 Å. The consolidated protein structure was subsequently taken into receptor grid generation panel for receptor lattice generation, as binding pocket information plays an important role in structure‐based drug designing. The binding pocket residues were gathered from the literature. The residues like PHE 140, GLY 143, CYS 145, HIS 163, HIS 164, GLU 166, GLY 189, and THR 190 were mainly responsible for the protein–ligand interaction for M^Pro^ and ACE2. The grid package was generated by selecting the centroid of the chosen residues (20 Å × 20 Å × 20 Å),, and then the grid constructed file was further used as input file for molecular docking studies.

**HIGHLIGHTS**

- Identified the potential bioactive compounds from *Ipomoea obscura* (L.) leaf extract.
- Molecular docking studies against ACE2 and M^Pro^ in SARS‐CoV genome.
- Observed urso‐deoxycholic acid, demeclocycline, tetracycline, chlorotetracycline and ethyl iso‐allocholate has potential viral inhibitory activity.
- Molecular interaction study claims that urso‐deoxycholic acid competitively binds with hACE2 and M^Pro^.

#### 2.5.2 Preparation of *I. obscura* (L.) chemical constitution

Eleven bioactive compounds from medicinal plant *I. obscura* (L.) were identified. The ligands were subjected into Ligprep to optimize low‐energy, three‐dimensional (3D) structure with proper chiralities for each molecular arrangement. It generated various structure isolated molecules with ionization states, tautomers, stereo chemistries, and ring conformations. The force field was optimized by OPLS3.

#### 2.5.3 *In silico* docking into therapeutic

Glide is a popular and reliable tool for docking investigations, and here we employed glide version 5.5 performing for docking studies. The refined target protein structure (PDB ID: 6LU7, 6M0J) was utilized as the receptor, and prepared bioactive compounds from medicinal plant *I. obscura* (L.) were docked with an active site of target proteins by the Glide XP model (Schrodinger, 2009). We analyzed the protein–ligand interactions, glide score, and energy by a Glide XP visualizer.

### 2.6 ADME properties assessment

Nowadays a failure rate of drug candidate increases in clinical stages due to undesired pharmacokinetics properties. The Absorption, Distribution, Metabolism, and Excretion (ADME) properties evaluation plays an important role in drug discovery. However, determination of ADME experimentally is time consuming and expensive process. Thus, most of the pharmaceutical companies spend more amount on increasing the success rate of drug candidate/s. Interestingly, pharmaceutical companies and research groups mainly depend upon computational approach for ADME calculation. Qikprop has been employed to evaluate the drug‐like properties of candidates (Lipinski, Lombardo, Dominy, & Feeney, 1997; QikProp, 2010) for instance, coefficient (QPlog*P* octanol/water), the water solubility (QPlogS), Lipinski's rule of five, gut blood–brain barrier (QPPCaco2), number of rotatable bonds, hydrophilic component of SASA, log IC50 value for blockage of K^+^ channels (QPlogHERG), percentage of human cell oral absorption, log *P* (water/gas), molecular weight, hydrogen bond donor, and hydrogen bond acceptor, which have been profiled for pharmacokinetics properties (Duffy & Jorgensen, 2009).

## 3 RESULTS AND DISCUSSION

**FIGURE 1:**
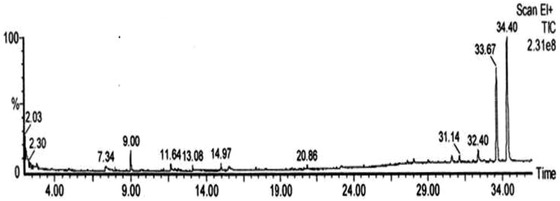
Gas‐chromatogram of ethanolic leaf extract of *I. obscura* (L.)

The GC‐MS is extensively used in medical, pharmaceutical, environmental, and forensic applications, comprising of two analytical techniques with single methodology for investigating a mixture of chemical compounds present in extract (Casuga, Agnes, & Corpuz, 2016; Rao, Asheervadam, Khalilullah, & Murti, 1989). The components present in ethanolic leaf extract of *I. obscura* (L.) were identified, and their obtained chromatogram is shown in Figure 1. Active principles with their retention time molecular formula, molecular weight (MW), and percentage composition in ethanolic leaf extract of *I. obscura* (L.) had been identified. Compound prediction was based on the NIST library such as lycopene (92.08%), 2‐cholestanone, 3‐phenyl‐ (76.15%), demeclocycline (17.53%), oleic acid (15.5%), cholestane‐3,5‐dichloro‐6‐nitro (11.08%), heptadeane, 9‐hexyl (7.30%), tetracycline (5.13%), octadecane, 3‐ethyl‐5‐(2‐ethylbutyl) (4.51%), cholortetracycline (4.15%), ethyl iso‐allocholate (3.54%), urso‐deoxycholic acid (3.23%) (see Tables 1 and 2).

**TABLE 1:** GC‐MS spectral analysis of ethanolic leaf extract of *I. obscura* (L.)

S. No.	Retention time	Name of the compound	Molecular formula	Molecular weight	Peak (%)
1	2.30	Heptadeane, 9‐hexyl‐	C_23_H_48_	324	7.30
2	7.34	Octadecane,3‐ethyl‐5‐(2‐ethylbutyl)‐	C_26_H_54_	366	4.51
3	9.00	Oleic acid	C_39_H_76_O_3_	592	15.5
4	11.64	Tetracycline	C_22_H_24_N_2_O_8_	444	5.13
5	13.08	Ursodeoxycholic acid	C_24_H_40_O_4_	392	3.23
6	14.97	Ethyl iso‐allocholate	C_26_H_44_O_5_	436	3.54
7	20.86	Cholortetracycline	C_22_H_23_CIN_2_O_8_	478	4.15
8	31.14	Cholestane‐3,5‐dichloro‐6nitro	C_27_H_45_CL_2_NO_2_	485	11.08
9	32.40	Demeclocycline	C_21_H_21_ClN_2_O_8_	464	17.53
10	33.67	2‐Cholestanone,3‐phenyl‐	C_33_H_50_O	462	76.15
11	34.40	Lycopene	C_40_H_56_	536	92.08

**TABLE 2:** Two‐dimensional chemical structures of the 11 bioactive compounds from *I. Obscura* (L.)

No	Name of the compound	Molecular formula
1	Heptadeane, 9‐hexyl‐	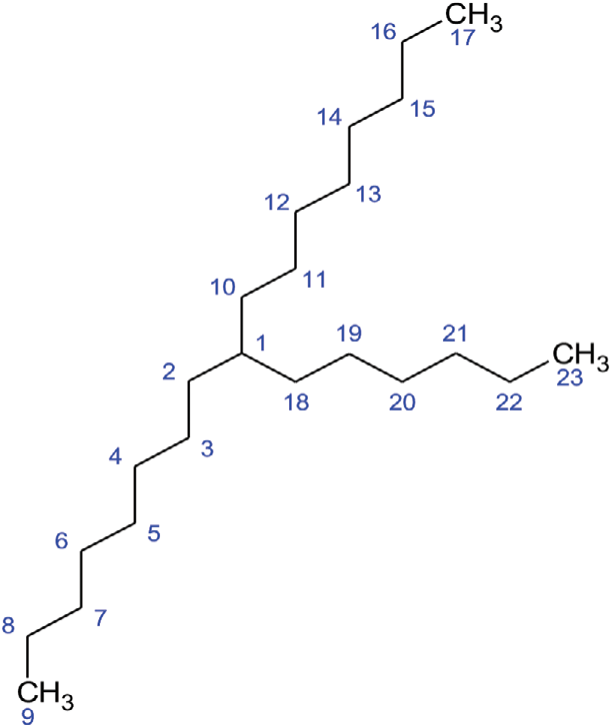
2	Octadecane,3‐ethyl‐5‐(2‐ethylbutyl)‐	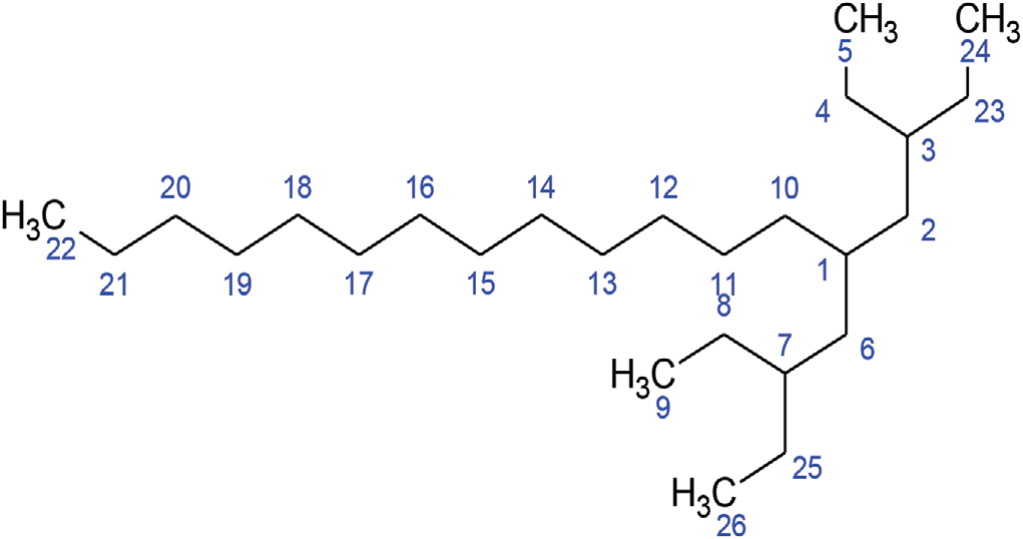
3	Oleic acid	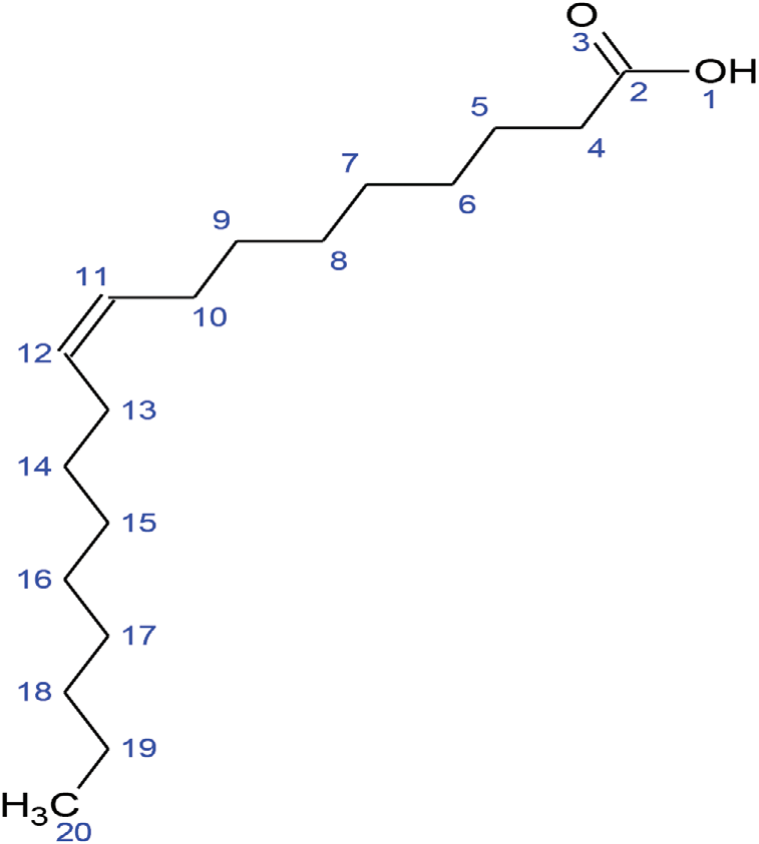
4	Tetracycline	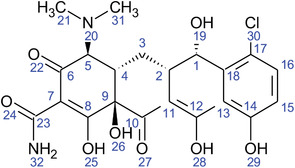
5	Ursodeoxycholic acid	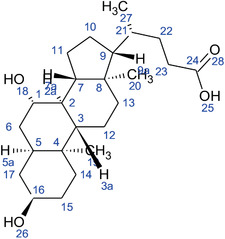
6	Ethyl iso‐allocholate	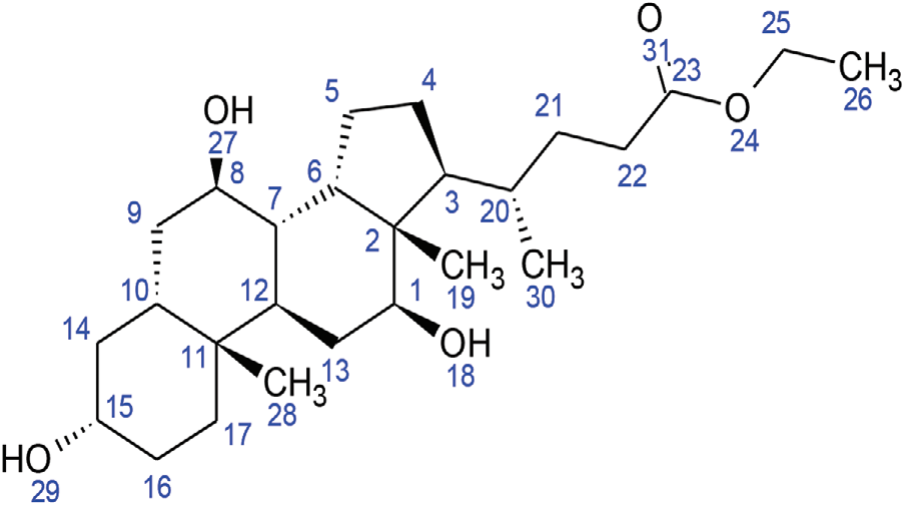
7	Cholortetracycline	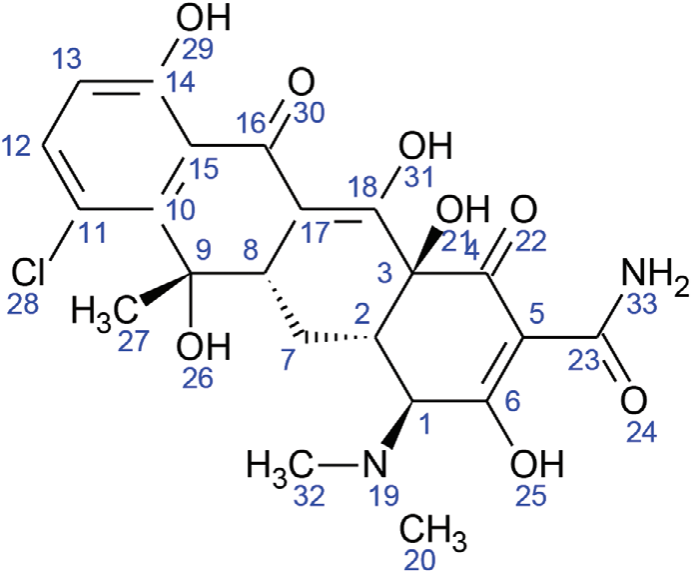
8	Cholestane‐3,5‐dichloro‐6nitro	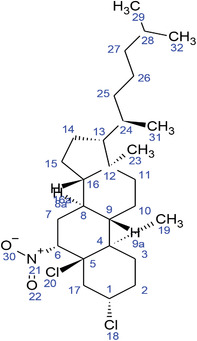
9	Demeclocycline	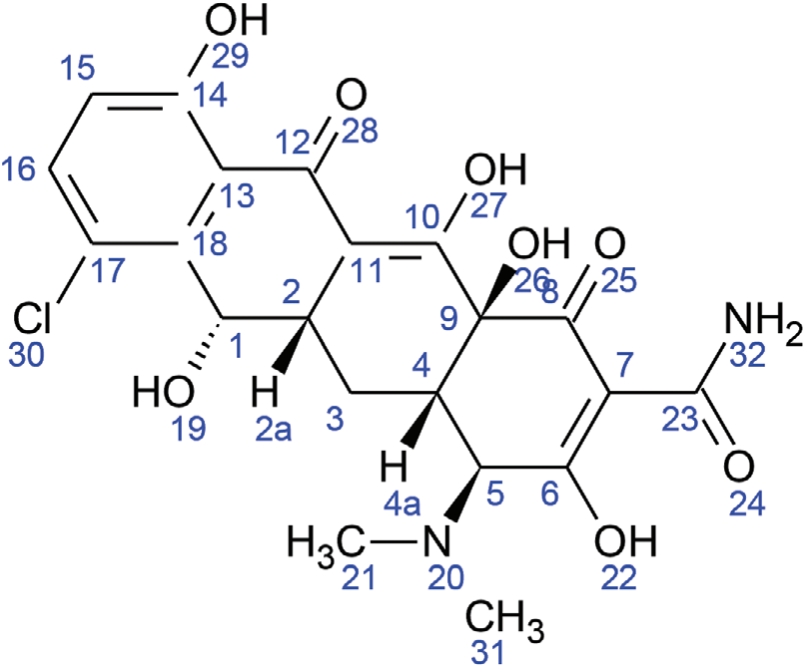
10	2‐Cholestanone,3‐phenyl‐	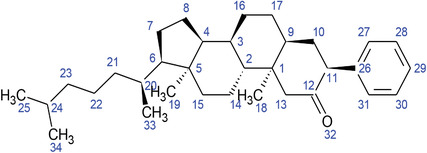
11	Lycopene	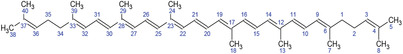

### 3.1 Docking simulation results of compounds present in *I. obscura* (L.) with ACE2 protein and M^Pro^

**FIGURE 2:**
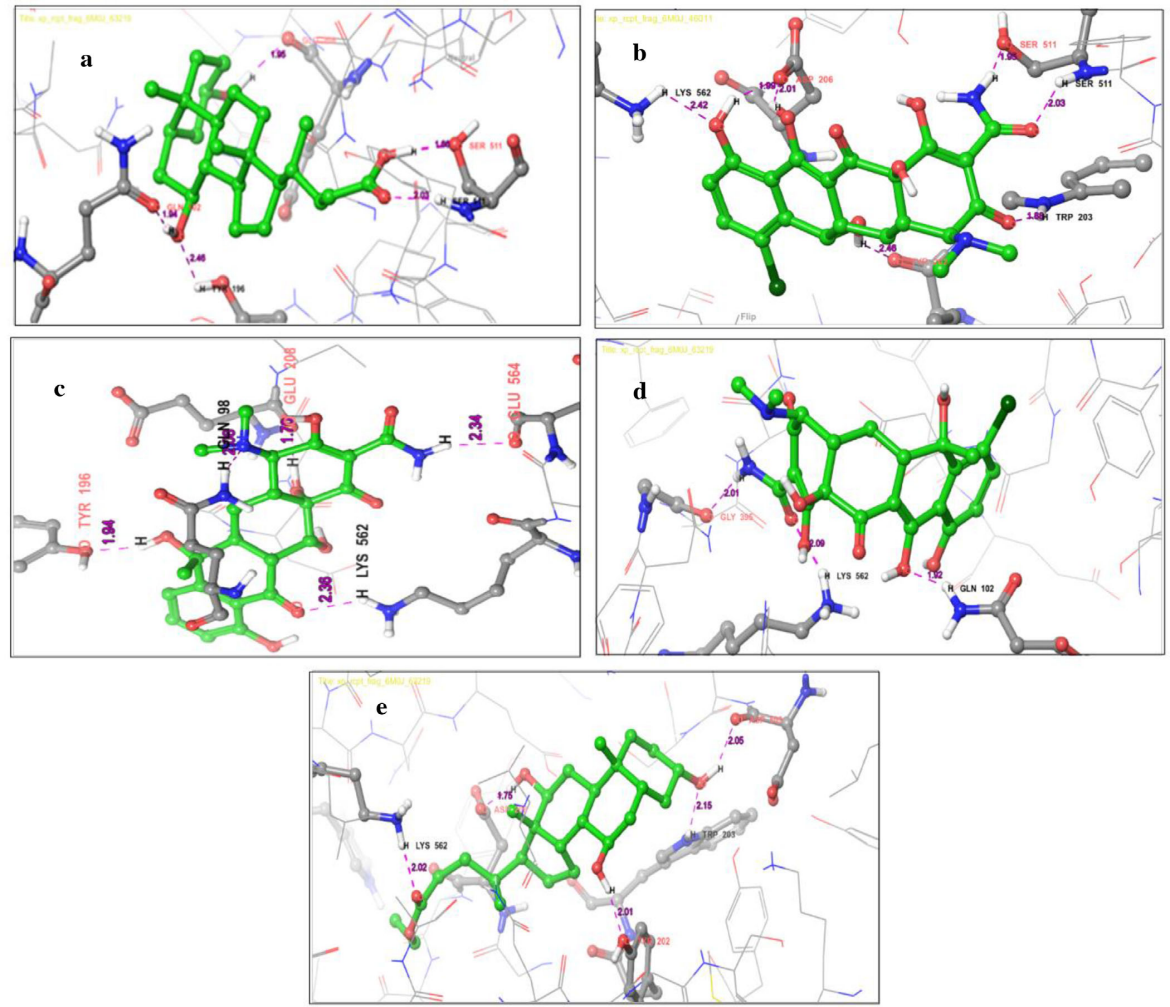
Two‐dimensional interaction representation of the top five hit compounds in the active site of the ACE2: (a) urso‐deoxycholic acid, (b) demeclocycline, (c) tetracycline, (d) chlorotetracycline, and (e) ethyl iso‐allocholate

**FIGURE 3:**
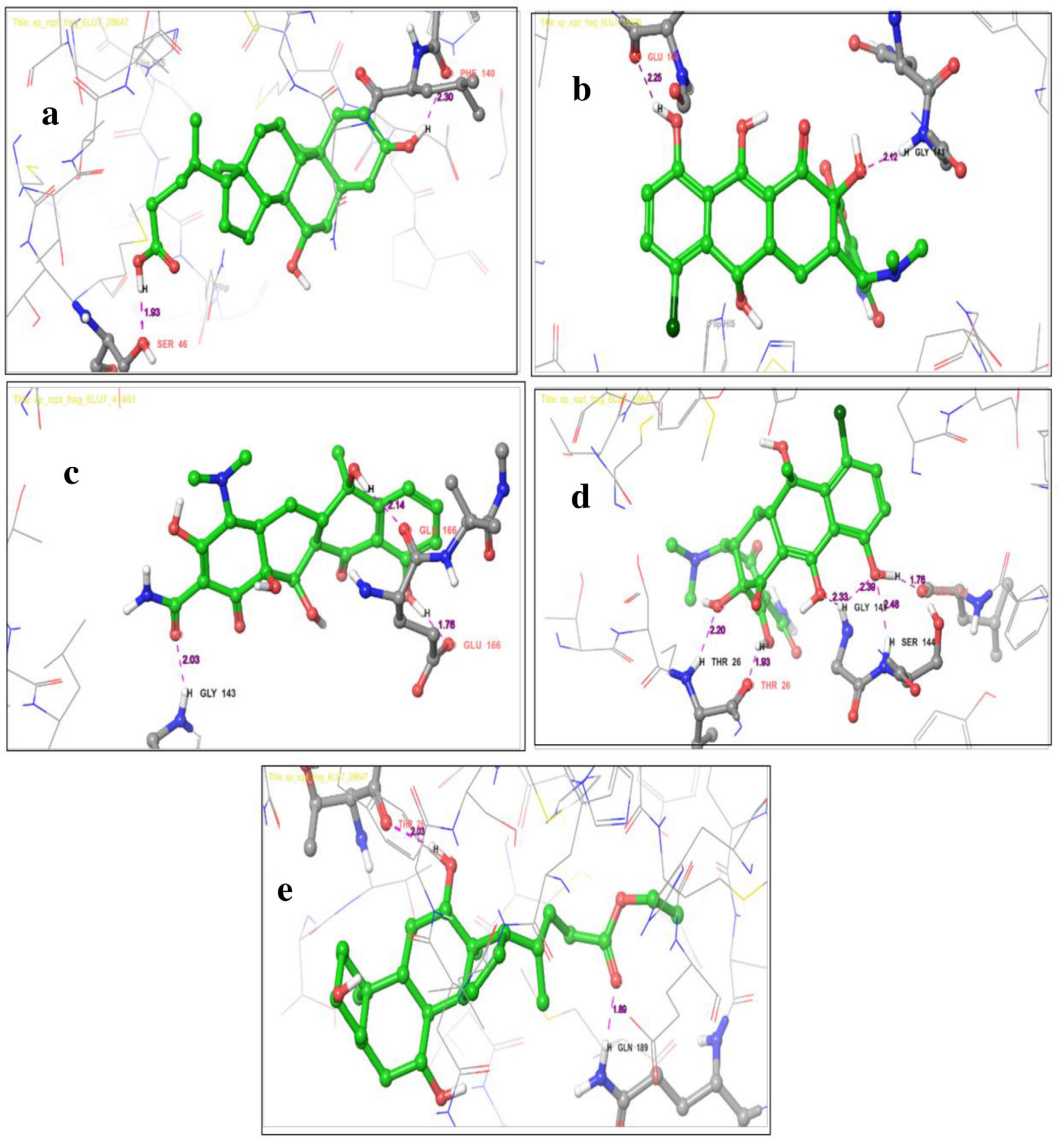
Three‐dimensional interaction representation of the top five hit compounds in the active site of the SARS‐CoV‐2 M^Pro^: (a) urso‐deoxycholic acid, (b) demeclocycline, (c) tetracycline, (d) chlorotetracycline, (e) Ethyl iso‐allocholate

Interaction exposed that the key residues SER 511, TYR 196, GLN 102, and GLU 208 involved in hydrogen bond interacting ACE2 with the bond distance of 2.03, 1.66, 2.46, 1.94, and 1.95 Å, respectively. TRP 203, TYR 202, ALA 99, TYR 510, and LEU 95 residues participated in hydrophobic interaction. The glide score −7.739 kcal/mol and glide energy −48.990 kcal/mol were computed and are shown in Table 3 and Figure 2. The 6LU7 is the M^pro^ found in the CoV associated with SARS and emerged as a potential drug target for COVID‐19 (Khan, Khan, Khan, Ahmad, & Ansari, 2020). Initially, we observed reference molecule N3 glide score and glide energy −6.633 kcal/mol and −82.592 kcal/mol, respectively. The following residues ASP 287, HIS 41, and GLU 166 held on hydrogen bond interacting M^pro^ with a bond length 2.27, 2.26, 2.22, and 2.10 Å and the following residues MET 49, CYS 44, VAL 186, CYC 145, PHE 181, LEU 141, TYR 54, and LEU 141 were involved in hydrophobic interaction with M^pro^. The urso‐deoxycholic acid had two hydrogen bond interactions with M^pro^. Backbone oxygen atoms in PHE 140 interact with the hydrogen atom of urso‐deoxycholic, and the hydrogen atom of urso‐deoxycholic interacts with a side chain oxygen atom of SER 46 with a bond length (2.30, 1.93 Å). MET 49, MET 165, CYS 145, and LEU 141 participated in the hydrophobic interaction that enhanced stability of the protein–ligand complex as shown in Figure 3. The glide score −7.111 kcal/mol and glide energy −46.632 kcal/mol were calculated (see Table 4).

**TABLE 3:** Glide extraprecision (XP) results between molecular docking of five hit molecules and ACE2 using Schrodinger 10.2

S. No	Compound name	Glide score	Glide energy	Number of hydrogen bonds	Interaction residues	Distance (Å)
1	Urso‐deoxycholic Acid	−7.739	−48.990	5	SER 511	2.03
					TYR 196	1.66
					GLN 102	2.46
					GLU 208	1.94, 1.95
2	Demeclocycline	−6.814	−62.708	7	ASP 206 (2)	1.99, 2.01
					TYR 202	2.46
					SER511 (2)	2.03, 1.95
					TRP 203	1.88
					LYS 562	2.42
3	Tetracycline	−5.809	−54.607	5	GLU 564	2.34,
					LYS 562,	2.36,
					GLN 98	2.08,
					GLU 208	1.70,
					TYR196	1.94
4	Chlorotetracycline	−5.405	−51.811	3	GLN 102	1.92
					LYS 562	2.09
					GLU 395	2.01
5	Ethyl iso‐allocholate	−4.818	−43.927	5	ASP 509	2.05
					TRP 203	2.15
					TYR 202	2.01
					ASP 206	1.75
					LYS 562	2.02

**TABLE 4:** Glide extraprecision (XP) results between molecular docking of five hit molecules and M^Pro^ using Schrodinger 10.2

S. No	Compound name	Glide score	Glide energy	Number of hydrogen bonds	Interaction residues	Distance (Å)
1	Urso‐deoxycholic acid	−7.11	−46.632	2	SER 46	1.93
					PHE 140	2.30
2	Demeclocycline	−6.807	−53.654	2	GLU 143	2.12
					GLU 166	2.25
3	Tetracycline	−5.949	−56.658	3	GLU 166 (2)	1.76
						2.14
4	Chlorotetracycline	−4.718	−40.084	6	THR 26 (2)	1.93
					GLU 143 (2)	2.20
					SER 144	2.33
					LEU 141	2.39
5	Ethyl iso‐allocholate	−4.416	−43.229	2	THR 26	2.03
					GLU 189	1.89

### 3.2 ADME properties prediction

The bioactive compounds were further assessed for their drug‐like behavior of ADME by use of QikProp. For the five bioactive molecules, the aqueous solubility (QPlog*S*) necessary for absorption and delivery of drug inside the human body range between −5.766 and −2.623, respectively. The percentage of human oral absorption for the bioactive compounds ranged from 23% to 95%. The predicted value of binding to human serum albumin (QPksha) fitted well within the acceptable range (∼ −0.637 to −0.060). The predicted blood/brain barriers were within the acceptable range (∼ −2.242 to −1.357). All the ADME properties are in adequate quality for solubility and permeability of cell membrane (Table 5): *S* in mol/L (acceptable range −6.5 to 0.5); percentage of human oral absorption (<25% is poor and >80% is high); prediction of binding to human serum albumin (acceptable range −1.0 to 1.5); prediction of brain/blood (acceptable range −3.0 to 1.2); the predicted rotatable bonds fit well with acceptable range 6–8; the predicted hydrophilic surface accessible solvent area (SASA) is under an acceptable range 158.858–314.842; molecular weight (<500 Da); hydrogen bond donor (<5); hydrogen bond acceptor (<10); predicted octanol/water partition coefficient log *p* (acceptable range −2.0 to 6.5).

**TABLE 5:** Predicted aqueous solubility

Ligand ID	Hydrophilic SASA	RB	QPlog*S*	Percent human oral absorption	QPlogKhsa	log BB	Molecular weight	HBD	HBA	QPlog (o/w)
Ursodeoxycholic acid	189.129	6	−4.96	81	0.44	−1.35	392.5	3	5.40	3.84
Demeclocyclin	314.842	7	−2.67	24	−0.27	−2.17	464.8	4	10.2	0.09
Tetracycline	298.331	7	−2.62	23	−0.12	−2.24	446.4	3	9.20	0.06
Chlorotetracycline	280.885	7	−2.84	27	−0.06	−1.90	478.8	4	9.25	0.06
Ethyl iso allocholate	158.858	8	−5.76	95	0.63	−1.43	436.6	3	7.10	3.82

RB, Rotatable Bond; BB, Blood Brain Barrier; HBD, Hydrogen Bond Donor; HBA, Hydrogen Bond Acceptor.

### 3.3 Molecular dynamics trajectory analysis

**FIGURE 4:**
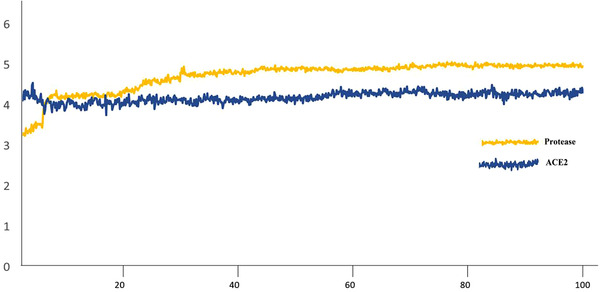
RMSD graph of SARS‐CoV‐2 M^Pro^ and ACE2–Urso‐deoxycholic acid for the 100‐ns simulation time

**FIGURE 5:**
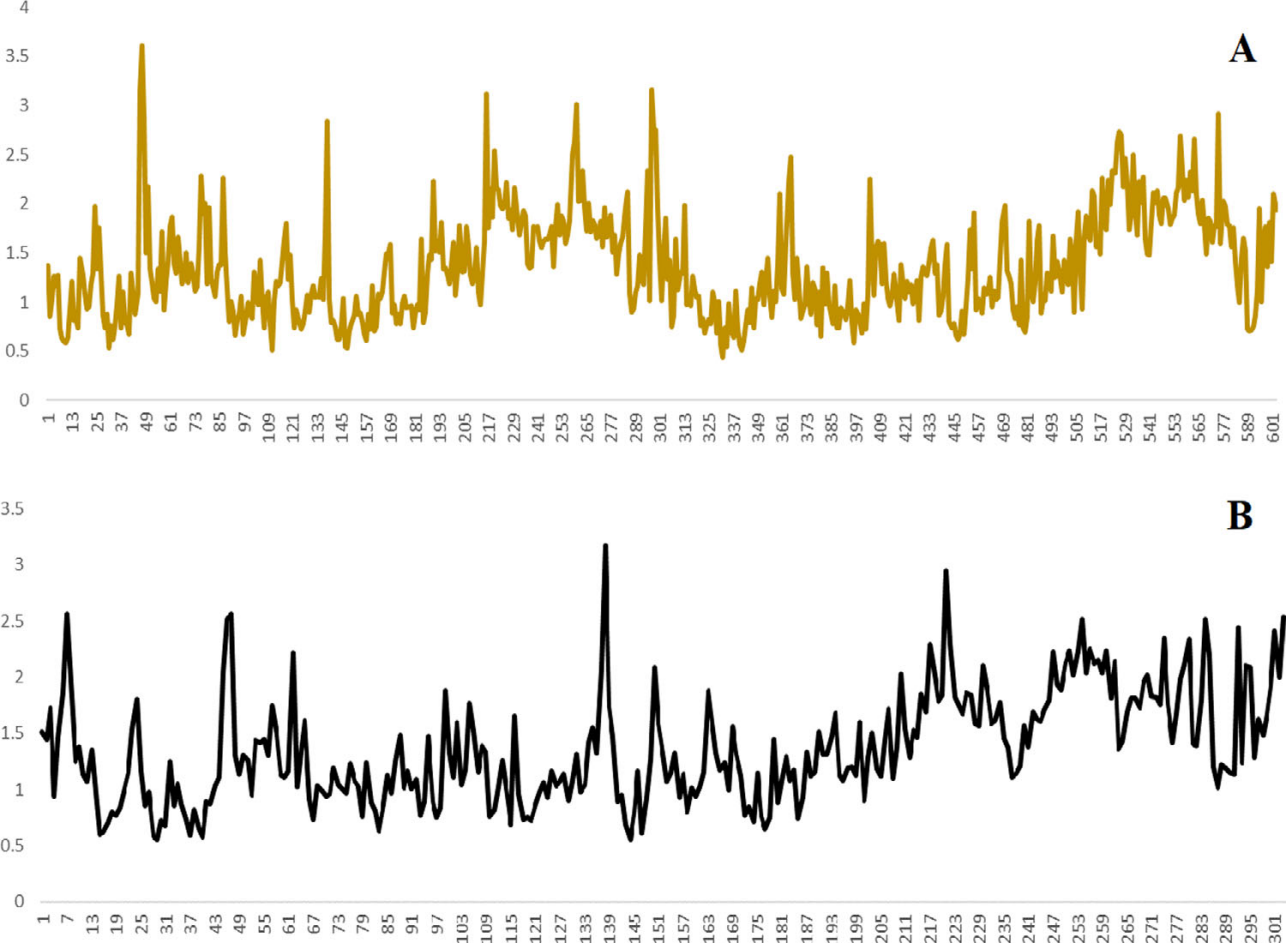
RMSF graph of SARS‐CoV‐2 M^Pro^ (a) ACE2 and (b) urso‐deoxycholic acid for the 100‐ns simulation time

The RMSD and RMSF graphs for ACE2 versus urso‐deoxycholic and M^pro^ versus urso‐deoxycholic are plotted in Figures 4 and 5. The M^pro^‐urso‐deoxycholic showed slight fluctuations at 5 than the entire simulation time. Initially, the RMSD of the main protease slightly fluctuated, the residues 48–138 were more stable, then slight fluctuation occurred between residues 139–143, and remaining residues were more stable during the entire simulation. The RMSD of the ACE2 showed slight changes in starting time of simulation and reached maximum 3.5 Å, but some fluctuations or changes occurred between the residues 38 and 58. This molecular dynamic study suggested that the complex of SARS CoV‐2 and ACE2‐ursodeoxycholic acids was more stable and produced increasingly reliable binding capabilities.

## 4 CONCLUSION

Medicinal value of plants offers protection and cure to human beings from various life‐threatening diseases. The existence of various bioactive compounds presents in plants provides evidence for the effective use against various ailments by conventional specialists. In the present work, phytochemicals were obtained from the leaf extract of *I. obscura* (L). by GC‐ MS analysis. It is noteworthy that the chemical compounds of *I. obscura* can inhibit the ACE2 protein and M^pro^ to block the viral receptivity. The molecular interaction study claims that urso‐deoxycholic acid from *I. obscura* (L.) efficiently binds with ACE2 and M^Pro^. Further, *in vivo* and *in vitro* methods are consequently recommended to explicate the molecular mechanism, toxicity of the active constituents, side effects, circulating level, and pharmacokinetic properties of urso‐deoxycholic acid to develop it as a potent drug against COVID‐19.

## CONFLICT OF INTEREST

The authors declare no competing financial interest.

## AUTHORS CONTRIBUTION

TK, EM, MA, and BB conceived and designed the experiments and wrote the paper. PSP, AM, AJ, and KBH contributed reagents and materials, performed the experiments, analyzed and interpreted the data, and wrote the paper. PS, AVA, KS, and VKG analyzed and interpreted the data and wrote the paper.

## References

[fft229-bib-0002] Aiping, W. , Yousong, P. , Baoying, H. , Xiao, D. , Xianyue, W. P. N , Jing, M. , … Taijiao, J. , (2020). Genome composition and divergence of the novel coronavirus (2019‐nCoV) originating in China. Cell Host Microbe, 27, 325–328. 32035028 10.1016/j.chom.2020.02.001 PMC7154514

[fft229-bib-0008] Andersen, K. G. , Rambaut, A. , Lipkin, W. I. , Holmes, E. C. , & Garry, R. F. , (2020). The proximal origin of SARS‐CoV‐2. Nature Medicine, 26, 450–452. 10.1038/s41591-020-0820-9 PMC7095063 32284615

[fft229-bib-0006] Anthony, R. F. , & Stanley, P. (2015). Coronaviruses: An overview of their replication and pathogenesis. Methods in Molecular Biology, 1282, 1–23. 25720466 10.1007/978-1-4939-2438-7_1 PMC4369385

[fft229-bib-0015] Bui, T. P. T. , Tran, T. A. M. , Nguyen, T. T. H. , Le, T. H. , Tran, T. H. , Huynh, T. P. L. , … Nguyen, T. A. N. (2020). Investigation into SARS‐CoV‐2 resistance of compounds in garlic essential oil. ACS Omega, 5(14), 8312–8320. 32363255 10.1021/acsomega.0c00772 PMC7123907

[fft229-bib-0026] Casuga, F. P. , Agnes, L. C. , & Corpuz, M. J. T. (2016). GC‐MS analysis of bioactive compounds present in different extracts of an endemic plant *Broussonetia luzonica* (Blanco Moraceae) leaves. Asian Pacific Journal of Tropical Biomedicine, 6(11), 957–961.

[fft229-bib-0004] Christophe, W. , & Pharm, D. (2002). Ethno pharmacology of medicinal plants: Asia and the Pacific (Vol. 1, p. 69). New York, NY: Humana Press,

[fft229-bib-0018] Dik‐Lung, M. , Daniel, S. C. , & Chung, H. L. (2011). Molecular docking for virtual screening of natural product databases. Chemical Science, 2, 1656‐1665.

[fft229-bib-0025] Duffy, E. M. , & Jorgensen, W. L. (2009). Prediction of properties from simulations: Free energies of salvation in Hexa decane, octanol, and water. Journal of American Chemical Society, 122, 2878–2888.

[fft229-bib-0011] Fatima, A. , & Florian, K. , (2020). SARS‐CoV‐2 vaccines: status report. Immunity, 52(4), 583–589. 32259480 10.1016/j.immuni.2020.03.007 PMC7136867

[fft229-bib-0020] Jin, Z. , Du, X. , Xu, Y. , Deng, Y. , Liu, M. , Zhao, Y. , … Yang, H. (2020). Structure of M^pro^ from COVID‐19 virus and discovery of its inhibitors. Nature, 582 (7811), 289–293. 32272481 10.1038/s41586-020-2223-y

[fft229-bib-0028] Khan, M. F. , Khan, M. A. , Khan, Z. A. , Ahmad, T. , & Ansari, W. A. (2020). Identification of dietary molecules as therapeutic agents to combat COVID‐19 using molecular docking studies. Computational Chemistry. (1). 10.21203/rs.3.rs-19560/v1

[fft229-bib-0016] Lafferly, M. F. W. (1989). Registry of mass spectral data, 5th ed. New York: Wiley.

[fft229-bib-0021] Lan, J. , Ge, J. , Yu, J. , Shan, S. , Zhou, H. , Fan, S. , … Wang, X. (2020). Structure of the SARS‐CoV‐2 spike receptor‐binding domain bound to the ACE2 receptor. Nature, 581(7807), 215–220. 32225176 10.1038/s41586-020-2180-5

[fft229-bib-0024] Lipinski, C. A. , Lombardo, F. , Dominy, B. W. , & Feeney, P. J. (1997). Experimental and computational approaches to estimate solubility and permeability in drug discovery and development settings. Advanced Drug Delivery Reviews, 23(1‐3), 3–25. 10.1016/s0169-409x(00)00129-0 11259830

[fft229-bib-0005] Markus, H. , Hannah, K. W. , Simon, S. , Nadine, K. , Tanja, H. , Sandra, E. , … Stefan, P. (2019). SARS‐CoV2 cell entry depends on ACE2 and TMPRSS2 and is blocked by a clinically proven protease inhibitor. Cell, 181, 1–10.

[fft229-bib-0012] Macchiagodena, M. , Pagliai, M. , & Procacci, P. , (2020). Identification of potential binders of the main protease 3CL^pro^ of the COVID‐19 via structure‐based ligand design and molecular modeling. Chemistry Physics Letter, 750, 137489. 10.1016/j.cplett.2020.137489 PMC7165110 32313296

[fft229-bib-0013] Mothay, D. , & Ramesh, K. V. , (2020). Binding site analysis of potential protease inhibitors of COVID‐19 using AutoDock. Virusdisease, 2, 1–6. 10.1007/s13337-020-00585-z PMC7195910 32363219

[fft229-bib-0014] Muralidharan, N. , Sakthivel, R. , Velmurugan, D. , & Michael Gromiha, M. , (2020). Computational studies of drug repurposing and synergism of lopinavir, oseltamivir and ritonavir binding with SARS‐CoV‐2 protease against COVID‐19. Journal of Biomolecular Structure Dynamics. Advance online publication. 10.1080/07391102.2020.1752802 32248766

[fft229-bib-0010] Othman, H. , Bouslama, Z. , Brandenburg, J. T. , da Rocha, J. , Hamdi, Y. , Ghedira, K. , Srairi‐Abid, N. , & Hazelhurst, S. (2020). Interaction of the spike protein RBD from SARS‐CoV‐2 with ACE2: Similarity with SARS‐CoV, hot‐spot analysis and effect of the receptor polymorphism. Biochemistry and Biophysics Research Communication, 527(3), 702–708. 10.1016/j.bbrc.2020.05.028 PMC7221370 32410735

[fft229-bib-0023] QikProp . (2010). QikProp, version 2.3. New York, NY: Schrodinger LLC.

[fft229-bib-0027] Rao, P. S. , Asheervadam, Y. , Khalilullah, M. , & Murti, V. V. S. (1989). A revised structure for the isoflavone lanceolarrin. Phytochemistry, 28, 957–958.

[fft229-bib-0001] Saravana Prabha, P. , Krishna Chaithanya, K. , Zenebe, H. , Nagaraju, B. , & Gopalakrishnan, V. K. , (2017). Isolation and identification of bioactive compound from *Ipomoea obscura* (L.) ker gawl. Journal of Pharmaceutical Research, 11(1), 10–14.

[fft229-bib-0019] Schrodinger LLC . (2009). Glide, version 5.5. New York, NY: Author.

[fft229-bib-0022] Schrodinger LLC . (2009). Protein preparation Wizard Maestro. New York, NY: Author.

[fft229-bib-0003] Shahina, A. (1994). Handbook of Arabian medicinal plants. Boca Raton, FL: CRC Press, 90.

[fft229-bib-0017] Stein, S. E. , (1990). National Institute of Standards and Technology (NIST) Mass Spectral Database and Software, Version 3.02. Gaithersburg, MD: NIST.

[fft229-bib-0007] Yong, Z. Z , & Edward, C. H. (2020). A genomic perspective on the origin and emergence of SARSCoV‐2. Cell, 181(2), 1–5. 32220310 10.1016/j.cell.2020.03.035 PMC7194821

[fft229-bib-0009] Yifei, X. , (2020). Unveiling the origin and transmission of 2019‐nCoV. Trends in Microbiology, 28(4), 239–240. 32155431 10.1016/j.tim.2020.02.001 PMC7126210

